# Economically Optimal Wheat Yield, Protein and Nitrogen Use Component Responses to Varying N Supply and Genotype

**DOI:** 10.3389/fpls.2019.01790

**Published:** 2020-02-25

**Authors:** William L. Pan, Kimberlee K. Kidwell, Vicki A. McCracken, Ronald P. Bolton, Monica Allen

**Affiliations:** ^1^ Nutrient Cycling, Rhizosphere Ecology Laboratory, Department of Crop and Soil Sciences, Washington State University, Pullman, WA, United States; ^2^ College of Agricultural, Consumer, and Environmental Sciences, Urbana, IL, United States; ^3^ School of Economic Sciences, Washington State University, Pullman, WA, United States

**Keywords:** protein, wheat, G*pc*-B1, nitrogen use efficiency, fertilizer, Mitscherlich, economics

## Abstract

Improvements in market value of hard red spring wheat (HRS, *Triticum aestivum L*.) are linked to breeding efforts to increase grain protein concentration (GPC). Numerous studies have been conducted on the identification, isolation of a chromosome region (*Gpc-B1*) of Wild emmer wheat (*Triticum turgidum* spp. *dicoccoides*) and its introgression into commercial hard wheat to GPC. Yet there has been limited research published on the comparative responsiveness of these altered lines and their parents to varied N supply. There is increased awareness that wheat genetic improvements must be assessed over a range of environmental and agronomic management conditions to assess stability. We report herein on economically optimal yield, protein and nitrogen use efficiency (NUE) component responses of two Pacific Northwestern USA cultivars, Tara and Scarlet compared to backcrossed derived near isolines with or without the *Gpc-B1* allele. A field experiment with 5 N rates as whole plots and 8 genotypes as subplots was conducted over two years under semi-arid, dryland conditions. One goal was to evaluate the efficacy of the *Gpc-B1* allele under a range of low to high N supply. Across all genotypes, grain yield responses to N supply followed the classic Mitscherlich response model, whereas GPC followed inverse quadratic or linear responses. The *Gpc-B1* introgression had no major impact on grain protein, but grain N and total above ground crop N yields demonstrated quadratic responses to total N supply. Generally, higher maximum grain yields and steeper rise to the maxima (Mitscherlich c values) were obtained in the first site-year. Tara required less N supply to achieve GPC goals than Scarlet in both site-years. Genotypes with *Gpc-B1* produced comparable or slightly lower Mitscherlich A values than unmodified genotypes, but displayed similar Mitscherlich c values. Target GPC goals were not achieved at economic optimal yields based on set wheat pricing. Economic optimization of N inputs to achieve protein goals showed positive revenue from additional N inputs for most genotypes. While N uptake efficiency did not drop below 0.40, N fertilizer-induced increases in grain N harvest correlated well with unused post-harvest soil N that is potentially susceptible to environmental loss.

## Introduction

Global wheat (*Triticum aestivum L*.) production and consumption continues to rise (USDA FAS, 2020) as wheat continues to be a major source of human calories and protein (Mondal et al., 2016). Wheat has been the dominant crop in the inland Pacific Northwest (iPNW) USA since farming began in the late 1800’s (Pan et al., 2017). Grain protein and N use responsiveness to N management in hard red wheats is well recognized (Belete et al., 2018), leading to specific unit N recommendations by wheat class (Koenig, 2005; Brown et al., 2005; Jones and Olson-Rutz, 2012; Franzen, 2018). A tremendous range in grain protein concentration (GPC) can be seen with variable N fertilizer management (Walsh et al., 2018; Beres et al., 2018). This experiment focused on recropped wheat in the transition zone of eastern Washington state, between wheat-fallow and continuous cropping agroecological zones. Recropping hard red spring wheat (HRS) after winter wheat is a strategy for crop intensification for diversifying the system and markets (Pan et al., 2017).

Economically optimal N supply is dictated by the shape of yield-protein and N use efficiency responses to N supply, the relative prices of wheat and N fertilizer, and the market premium:discount prices for GPC of hard red wheat (Baker et al., 2004). In the U.S., hard red wheat grain price premiums and discounts are often assessed at the grain elevator, based on GPC. Bekkerman (2018) used differences between prices for hard red spring wheat futures contracts on the Minneapolis Grain Exchange (MGEX) and hard red winter wheat futures contracts on the Kansas City Board of Trade (KCBT) to study how the market values protein. He found that the long-run average spread is approximately $23.15/Mg. These economic analyses indicate that building grain protein through N fertilization is not always profitable, however, depending on external factors such as the cash price of wheat, availability of high-protein wheat, and the cost of N fertilizer. As a result, improving grain protein to enhance end-use quality has long been a breeding goal in wheat breeding programs.

Improvements in wheat production and quality, and improved water and nutrient use can be achieved through integrated, environmentally-targeted cultivar selection and management efforts (Hatfield and Walthall, 2015). DePauw and Townley-Smith (1988) determined that environment and management typically overshadow genetic effects on GPC. While GPC has long-been recognized as an important grain end-use quality attribute and economic market factor, breeding improvements in wheat grain concentration has progressed slowly (Carter et al., 2012; Tabbita et al., 2017).

Wheat genetic heritability of nitrogen use efficiency (NUE) physiological traits that contribute to high NUE and grain protein (Hawkesford, 2012), particularly for low N environments, has been reviewed by Dawson et al. (2008). For example, genetic variation for wheat N uptake (Johnson et al., 1967) and translocation efficiency (Cox et al., 1986) are well established. In addition, genetic variability for post-anthesis N uptake was early recognized (Clarke et al., 1990). Early wheat breeding efforts connected high grain protein with genetic improvement of nitrate reductase activity, N uptake and translocation of the hard red winter wheat cultivar Lancota (Johnson and Mattern, 1968). Loffler and Busch (1982) correlated grain yield with harvest index and N harvest index of hard red spring wheat varieties, but GPC was negatively correlated with harvest index, and not significantly correlated with N harvest index.

Avivi (1978) identified a gene associated with high GPC in wild emmer wheat (*Triticum turgidum* spp. *dicoccoides).* Joppa and Cantrell (1990) substituted chromosomes from the wild emmer wheat into the durum wheat cultivar Langdon, producing high GPC lines. A genomic region was mapped as a QTL in a recombinant inbred line of this cultivar (Joppa et al., 1997) and then mapped as a single Mendelian locus designated as DIC *Gpc-B1* (Olmos et al., 2003). Introgression of this region into tetraploid and hexaploid wheat increased GPC (Joppa et al., 1997; Mesfin et al., 2000, Distelfeld et al., 2007).

Tabbita et al. (2017) reviewed 25 studies conducted over 10 years characterizing the allelic variation of *Gpc-B1* and its effects on wheat yield and quality. Studies were conducted over a globally diverse range of wheat genetic backgrounds and environments. A few studies linked *Gpc-B1* related increases in GPC to accelerated leaf senescence (Uauy et al., 2006; Carter et al., 2012) and more efficient N remobilization from leaves (Kade et al., 2005). Yet, the surveyed papers generally lacked examination of how genotypes with *Gpc-B1* respond to a wide range of N supply. Moll et al. (1982) defined a statistical evaluation of genotypic variation of nitrogen use efficiency (NUE) and its mathematical relationship to NUE components: N uptake efficiency (NUPE; crop N/N supply) and N utilization efficiency (NUTE; grain yield/crop N). Their analysis of corn genotypes suggested that variation in N utilization contributed to genetic differences at low N supply, whereas variation in N uptake efficiency was the major source of NUE genetic variation at high N supply. Dawson et al. (2008) suggested the possibility of developing wheat cultivars with high NUE in low N environments, when high remobilization of leaf N into grain will be critical. Therefore, the objectives of this research were to determine whether introgressing *Gpc-B1* into two commercial hard red spring wheat cultivars would improve yield, protein and NUE component responsiveness over a wide range of N supply, while reducing economically optimal N requirements.

## Materials and Methods

### Experimental Conditions

This experiment was conducted over two years near Dusty in 2004 and Endicott Washington, U.S in 2005 under semi-arid, dryland conditions. The soil at Dusty, WA was an Onyx silt loam (coarse-silty mixed mesic Cumulic Haploxerolls) soil type, that received 378 mm of annual precipitation. The soil at Endicott, WA was an Athena silt loam (fine-silty mixed mesic Pachic Haploxerolls) that received 424 mm of precipitation. Genotypes were direct seeded into winter wheat stubble from fields yielding 4,200 and 3,600 kg ha^-1^ in the first and second site-years, respectively. Fall pre-fertilization soil sampling consisted of triplicate samples taken to 120 cm depths in each replicate block, and 30 cm depth increments were composited by replicate for determination of 1 M KCl exchangeable NH_4_^+^-N and NO_3_^–^N. Samples were taken with a tractor (John Deere 5425, Moline, IL)-mounted hydraulic probe (Giddings, Windsow, CO). Additional general soil fertility tests were performed on the 0-30 cm samples. Samples were stored at -15.6°C prior to inorganic N determination using flow injection autoanalysis (Quickchem 8000 Series FIA+ system, Lachat Instruments, Loveland, CO). Soil nitrate-N was measured at each depth, and soil ammonium-N was measured in the 0-30 cm samples. Net mineralization was estimated as only 17 kg N ha^-1^ following winter wheat stubble, and when added to the residual mineral N resulted in estimated soil N supplies of 46 and 39 kg N ha^-1^ in the first and second site-years, respectively.

### Plant Germplasm and DNA Marker Analyses

Recurrent parents included the hard red spring wheat cultivars Scarlet (Kidwell et al., 1999), which was developed for the semi-arid region of Eastern Washington and Tara 2002, herein referred to as Tara (Kidwell et al., 2002), which was released for production in the high rainfall regions of the PNW. The donor parents were hard red spring wheat cultivar Glupro and ND683 from North Dakota (Mesfin et al., 1999; Khan et al., 2000). The high GPC region was incorporated into Scarlet and Tara using DNA markers to select for the presence of the region (Carter et al., 2012). At the time, Scarlet was a high yielding hard red spring wheat cultivar in the production region (Burns, 2002), and Tara was released based on its improved yield potential and superior end-use quality. The goal was to recover lines nearly identical (near isolines) to Scarlet and Tara with the addition of the high GPC segment from Glupro. The HRS wheat cultivar Glupro was used as the donor parent for the DIC *Gpc-B1* allele. Isolines included three BC_5_F_5_ marker-assisted backcross (MAB)-derived genotypes with the DIC *Gpc-B1* allele and three with the recurrent parent allele at the *Gpc-B1* locus. The backcross introgression process using DNA marker analysis to assay for the presence of the DIC *Gpc-B1* was described by Carter et al. (2012). The eight genotypes evaluated in this study were Tara, 3512-26T, 3586-6G/T, 3512-1G, Scarlet, 1519-16S, 1553-25G, 1584-12G where crosses with *Gpc-B1* have the letter G behind the cultivar; T or S indicates a near isoline of Tara or Scarlet and G/T represents the heterogeneous population.

### Experimental Design

A split-plot, randomized complete block field experiment with five fertilizer nitrogen (Nf) rates (0, 45, 90, 135, 179 kg N ha^-1^) as whole plots randomized in four replicate blocks. Eight genotypes were randomized as subplots within each N rate main plot. A basal fall application of 45 kg urea-N ha^-1^ was topdressed over all N fertilized plots and the remaining urea N fertilizer was applied in spring at planting, 10 cm below the seed. Row spacing was 18.5 cm in plots with 2.1 m x 12.2 m dimensions. Spring wheat lines were planted in late March at a 5 cm depth at a seeding rate of 78 kg ha^-1^ using a no-till drill (Fabro Ltd., Swift Current, SK, Canada).

### Sampling and Data Collection

Two, 1-m rows of plant material were hand-harvested at physiological maturity in early August. Grain was harvested a week later with a small-plot combine (Wintersteiger Inc., Salt Lake, UT, USA), After collection, samples were dried at 65°C, heads were threshed and the grain was dried, weighed, and ground with a Udy mill (Udy Corp., Fort Collins, CO) to <0.5 mm. The stover was ground <2 mm Wiley mill (Thomas Scientific, Swedesboro, NJ) for nitrogen analysis. Plant samples were evaluated for nitrogen, sulfur and carbon with the above ground biomass and grain analyzed separately using a dry combustion analyzer (Leco Corp., St. Joseph, MI, USA). The GPC was calculated as GPC = grain N (g/100g) * 5.7.

### Data Analysis

All data were subjected to analysis of variance using PROC MIXED procedure to compare means of the dependent variables in this study (SAS Institute Inc., 2013).

Replicate data of yield response to total N supply in individual site years were best fitted to the Mitscherlich growth factor response model using Sigmaplot (Systat Software, Inc., San Jose, CA). The Mitscherlich yield response to total N supply model (Pan et al., 2016) is defined as:

(1) $$\text{Y}=\text{A}\times (1–10^{–\text{c(X)}})$$

Where: Y = Gw, grain yield; X = Ns, N supply; A = maximum yield; c = efficiency constant.

Grain protein concentration (GPC), grain N yield, and above-ground crop N responses to N supply were all best-fitted with quadratic equations:

(2) $$\text{Y}=\text{a}+\text{b X}+{\text{c X}}^{2}$$

Total N supply estimated as described below (Huggins and Pan, 1993):

(3) Total N supply (Ns)=(pre−plant, root zone inorganic soil N)+[estimated net mineralized N (Koenig, 2005)]+(fertilizer N).

The regression analyses for the Mitscherlich-modeled yield and the quadratic-modeled crop N responses included virtual observations of zero yield and zero crop N at zero N supply, added for each genotype x N supply block replicate.

N use efficiency, grain N harvest efficiency and their components were defined (Moll et al., 1982) using grain weight (Gw), above-ground crop N uptake (Nt), and grain N (Ng) as the following ratios:

N use efficiency (NUE) = Gw/Ns

N uptake efficiency (NUPE) = Nt/Ns

N utilization efficiency (NUTE) = Gw/Nt

Nitrogen harvest index (NHI) = Ng/Nt

Nitrogen harvest efficiency (NHE) = Ng/Ns

where:

(4) $$\begin{array}{l} \text{Gw}/\text{Ns}=({\text{N}}_{\text{t}}/\text{Ns})\times (\text{Gw}/\text{Nt})=\\ (\text{Nt}/\text{Ns})\times (\text{Gw}/\text{Ng})\times (\text{Ng}/\text{Nt}),\text{ and} \end{array}$$

(5) Ng/Ns=(Nt/Ns)×(Ng/Nt)

These use efficiencies and components were statistically analyzed for main effects of genotype, N rate, year, and genotype × N rate.

### Economic Optimization of N Inputs

Mitscherlich equations (Eq. 1) identified c and A values for each genotype’s response to N supply in each year. These response models were then used with a fixed N and grain prices to obtain initial estimates of economically optimal N rates (EONR), supply (EONS), yield (EOY) and the corresponding unit N requirement (UNR) according to Fiez et al. (1995) as

(6) UNR=(EONS/0.01)×EOY

with no initial consideration of market valuation of protein. Economic optimal nitrogen rates (EONR) and total nitrogen supplies (EONS) required to achieve economic optimal grain yields (EOY) were determined by plotting a constant value (i.e. current market prices) of fertilizer N inputs (X$) at US $1.03 (kg Nf) ^–1^ vs. wheat grain (Y$) at $0.23 (kg grain) ^–1^ where dY$/dX$ = 0.23/1.03. This current market price was set as the base grain price for wheat at 140 g protein kg^-1^, which is a typical target GPC for U.S. hard red spring wheat. Net revenue is defined here as revenue over Ns cost at the optimum. Price discounts for low protein wheat were then applied to determine the adjusted economic value of grain produced at the initial EONS. Since none of the genotypes achieved this target GPC at EONS the grain prices were then adjusted with discounts, $0.009 kg^-1^ subtracted from the base price for each 2.5 g protein kg^-1^ below 140 g protein kg^-1^. The net revenue was then determined from the amount of additional N fertilizer required above the EONS to achieve the market target of 140 g protein kg^-1^. Premiums ($0.009 kg^-1^) were also added to the base wheat price (reported at 140 g protein kg^-1^) for each 2.5 g above 140 g kg^-1^ protein achieved when N supply was increased above that required to achieve 140 g protein kg^-1^.

Net revenue to farmers, assuming fertilizer N rate was the only varying management variable, was calculated as

(7) Net Revenue=(Gw×proteinbased grain price)−(Nf×N fertilizer price).

## Results

### Grain Yield, Grain N Yield, Crop N, and GPC Responses

**Figure 1:**
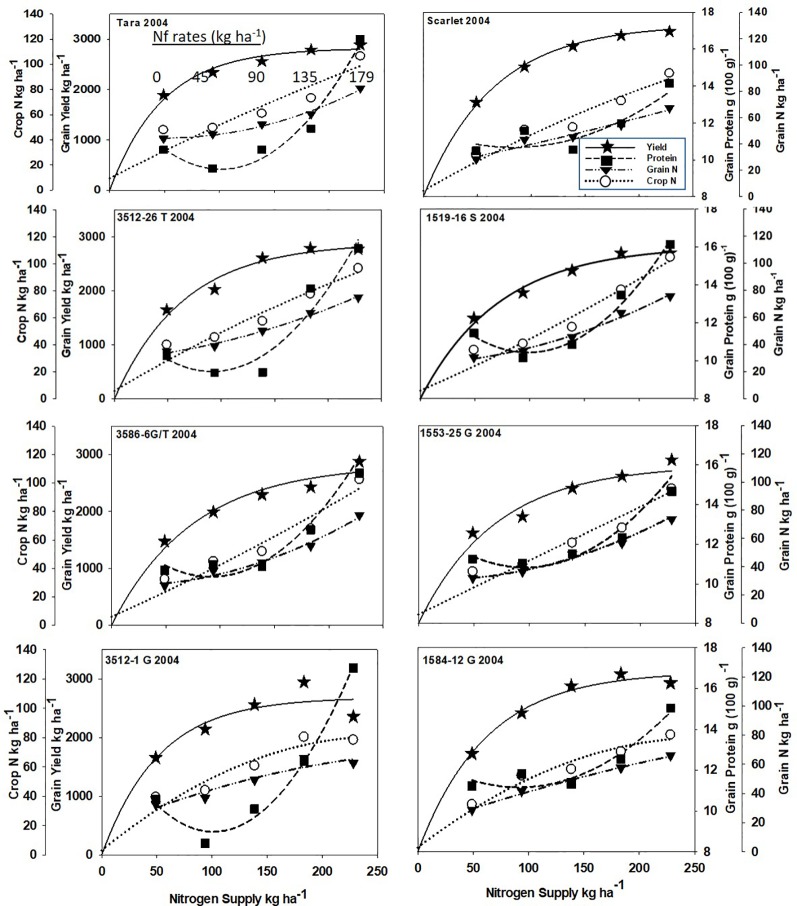
Grain yield, grain N concentration, grain N yield, and crop N responses to increasing N supply at Dusty WA in 2004 of HRS cultivar Tara and its derivatives 3512-26T, 3586-6G/T, 3512-1G; HRS cultivar Scarlet and its derivatives 1519-16S, 519-16S, 1553-26G and 1584-12G. Symbols represent means of 4 N rate replicates. Regression coefficients of responses modeled on entire datasets of each dependent variable are presented in **Tables 1** and **S1**.

**Figure 2:**
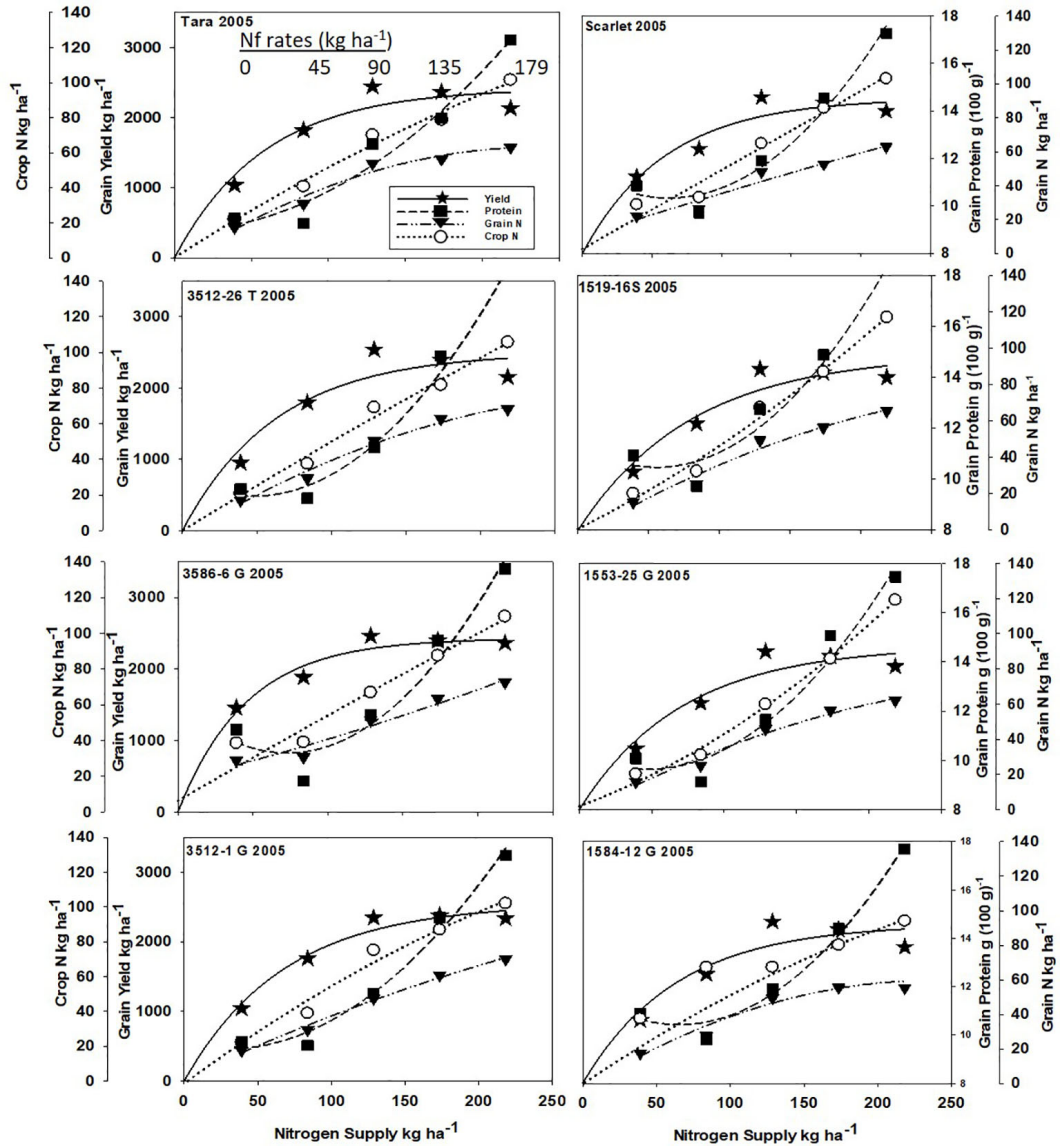
Grain yield, grain N concentration, grain N yield, and crop N responses to increasing N supply at Endicott WA in 2005 of HRS cultivar Tara and its derivatives 3512-26T, 3586-6G/T, and 3512-1G; HRS cultivar Scarlet and its derivatives 1519-16S, 1519-16S, 1553-26G and 1584-12G. Symbols represent means of 4 N rate replicates of each dependent variable. Regression coefficients of responses modeled on entire datasets are presented in **Tables 1** and **S1**.

Increased N supply significantly increased grain yield with a diminishing slope best represented by the Mitcherlich model, and it also increased GPC by inverse quadratic functions in both site-years (**Figures 1** and **2**; **Tables 1** and **S1**). The Mitscherlich efficiency coefficient “c”, which describes the steepness of the approach to maximum “A” did not statistically vary among genotypes or by site-year (**Table 1**). Supplying fertilizer N up to an additional 179 kg N ha^-1^ enabled us to establish maximum grain yields not limited by N supply in both site-years, whereas excess N continued to increase GPC past the point of maximum yield. Modeled grain yield plateaus (A values) in the first site-year ranged from 2651to 2889 kg ha^-1^, whereas yield plateaus in the second-site year were significantly lower based on pair-wise t tests, ranging from 2310 to 2497 kg ha^-1^ (**Figures 1** and **2**; **Table 1**). Supplying the lowest N rate input, 45 kg N ha^-1^, increased yield compared to the no N fertilizer control, while only maintaining or decreasing GPC (**Figures 1** and **2**). The increase in GPC was steeper once optimal grain yield was achieved, as GPC levels of 16 to 17 g (100 g)^-1^ were reached at the highest N supply (**Figures 1** and **2**).

**Table 1:** Mitscherlich model correlation coefficients, A and c parameters with their standard errors for yield response to N supply of all genotypes, shown in **Figures 1** and **2**.

Genotype	r^2^	A	*SD*	c	*SD*	EONS	EOY	EONR	UNR
**A. Dusty, 2004**										
1519-16 S	0.94	2827	146	0.0060	0.0009	157	2503	108	6.26
1553-25 G	0.89	2801	198	0.0062	0.0013	153	2487	104	6.17
1584-12 G	0.90	2651	147	0.0071	0.0013	139	2377	90	5.84
Scarlet	0.91	2802	153	0.0067	0.0012	147	2511	98	5.85
3512-1 G	0.76	2688	220	0.0084	0.0026	127	2456	78	5.16
3512-26 T	0.91	2889	167	0.0067	0.0012	149	2598	100	5.73
3586-6G/T	0.89	2829	211	0.0058	0.0012	160	2494	111	6.40
Tara	0.91	2824	121	0.0090	0.0015	124	2608	75	4.76
Site-year means		2789	171	0.0070	0.0014	143	2510	104	5.71
**B. Endicott, 2005**									
1519-16 S	0.92	2416	175	0.0054	0.0010	153	2056	114	7.45
1553-25 G	0.92	2326	151	0.0061	0.0011	141	2007	102	7.05
1584-12 G	0.91	2310	130	0.0068	0.0013	133	2024	94	6.59
Scarlet	0.84	2289	176	0.0073	0.0018	128	2022	89	6.33
3512-1 G	0.95	2548	121	0.0064	0.0009	144	2244	105	6.43
3512-26 T	0.89	2497	168	0.0069	0.0014	137	2216	98	6.20
3586-6G/T	0.90	2430	116	0.0095	0.0017	113	2225	74	5.08
Tara	0.92	2423	124	0.0075	0.0013	129	2164	90	5.98
Site-year means		2405	145	0.0070	0.0013	134	2126	95	6.30

Economic optimal N supply (EONS), yield (EOY), N rate (EONR), and Unit N requirement (UNR) were estimated initially using a base wheat price of $0.23/kg grain without protein discounts/premiums.

Analysis of variance revealed site-year, N rate and genotype were significant for all crop parameters except crop N, which was not different across genotypes (**Table S2**). No significant interactions were detected between N rate and genotype. Backcross derived near isolines with the *Gpc-B1* region did not have significantly higher GPC averages than their recurrent parent at the economically optimal N supply and yield when the grain protein premium was not considered (**Table 2**).

**Table 2:** Grain protein and net revenue generated from EONS and yields described in **Table 1**, and higher EONS required to generate higher net revenues calculated when meeting 14 and 15 g (100 g)^-1^ protein goals as protein price premiums are accounted.

**Genotype**	Varying Protein Levels at EONS				14 g (100 g)^-1^ Protein Level				15 g (100 g)^-1^ Protein Level		
	EONS	**Yield**	**Protein**	**Revenue**	**EONS**		**Yield**	**Revenue**	**EONS**	**Yield**	**Revenue**
	**kg/ha**		**g (100 g)^-1^**	**$/ha**	**kg/ha**			**$/ha**	**kg/ha**		**$/ha**
**A. Dusty, 2004**											
1519-16 S	157	2503	12.8	438	175		2576	468	188	2618	488
1553-25 G	153	2487	12.6	432	182		2593	465	198	2635	483
1584-12 G	139	2377	11.7	403	208		2563	431	228	2587	441
Scarlet	147	2511	10.3	391	315		2780	375	339	2787	378
3512-1 G	127	2456	10.9	414	206		2638	451	221	2650	463
3512-26 T	149	2598	12.8	465	174		2691	496	190	2735	514
3586-6G/T	160	2494	11.4	399	210		2658	452	224	2688	469
Tara	124	2608	10.8	447	209		2787	483	223	2797	496
**B. Endicott, 2005**											
1519-16 S	153	2056	13.4	344	163		2096	360	177	2149	377
1553-25 G	141	2007	12.6	330	163		2089	358	176	2129	374
1584-12 G	133	2024	11.6	323	179		2170	360	193	2198	372
Scarlet	128	2022	11.7	330	172		2161	365	186	2188	378
3512-1 G	144	2244	11.8	363	185		2381	403	200	2415	417
3512-26 T	137	2216	11.7	361	174		2339	405	186	2368	420
3586-6G/T	113	2225	11.4	382	160		2357	422	173	2375	435
Tara	129	2164	12.4	373	157		2262	403	172	2299	418

### Crop N and Grain N Yields

Crop and grain N responses to increasing N supply were best fitted by quadratic response functions (**Figures 1** and **2**; **Table S1**). Crop N functions were near linear, as illustrated by larger, linear coefficients that were more frequently significant than the smaller, more frequently non-significant quadratic coefficients (**Table S1**). In comparison, grain N accumulated with lower slopes (**Figures 1** and **2**) over the range of N rates, illustrating greater proportion of crop N stored in the straw with increasing N supply.

### Economic Optimization

Mitscherlich response functions were initially used with fixed N and grain prices to estimate economically optimal N rates (EONR), supply (EONS), yield (EOY), and the corresponding unit N requirement (UNR), all initially ignoring market valuation of protein (**Table 1**). While EOYs were lower in the second vs. first site-year, the EONS values were only slightly lower, thus resulting in higher UNRs in the second site-year. Derivatives of Tara and Scarlet generally exhibited higher EONS and UNR than their parents in both years. Nevertheless, 3586-6 G/T was an exception in the second site-year, exhibiting lower EONS and UNR than Tara.

Since high protein goals were not achieved with the initial EONS that was estimated without regard to protein premiums or discounts, we used the Mitscherlich yield and quadratic GPC functions to assess the net revenues obtained with additional N fertilizer additions beyond the initial EONS that elevated both yield and GPC (**Table 2**). While the higher N fertilizer investment per unit yield did not pay off for Scarlet in the first site-year due to a more gradual increase in GPC beyond the EONS required to achieve the initial EOY, overall net revenues of $25 and $ 37 ha^-1^ were obtained with increased Ns to achieve 14 and 15 g protein (100 g)^-1^ for Scarlet and its derivatives averaged over both site-years (**Table 2**). In contrast, higher net revenues of $39 and $54 ha^-1^ were obtained as Ns was increased to achieve 14 and 15 g protein (100 g) ^-1^ for Tara and its derivatives averaged over both site-years. Comparing N supply required to achieve these protein goals of the base cultivars in the high yielding first site-year, Scarlet required 315 and 339 kg Ns ha^-1^, while Tara only required 209 and 223 kg Ns ha^-1^ (**Table 2**). Similarly in the lower yielding second site-year, Scarlet required 172 and 186 kg Ns ha^-1^, while Tara only required 157 and 172 kg Ns ha^-1^ to achieve protein goals of 14 and 15 g protein (100 g)^-1^, respectively.

### NUE and Components

Analysis of variance revealed site-year and N rate effects were significant for NUE, NUPE, and NUTE, but genotype only affected NUE and NHI (**Table S2**). No significant interactions were detected between N rate and genotype for NUE and its components. The interactions between genotype and N rate were largely non-significant, so main effects of genotype (**Table 3**) and N rate (**Table 4**) are presented. Tara exhibited higher NUE and NHE than its derivatives in the first site-year due to higher N uptake efficiency rather than higher NHI (**Table 3**). Yet in all other comparisons, the advanced lines were not significantly different than either parents for both site-years (**Table 3**). One exception was 3586-6 G/T in the second site-year was higher than its parent Tara in NUE and grain N accumulation efficiency, due to higher N uptake efficiency.

**Table 3:** Genotypic means averaged over all N rates for N utilization (NUTE), N uptake (NUPE), nitrogen use efficiency (NUE), grain N harvest efficiency (NHE), and N harvest index (NHI).

Genotypes	NUTE	NUPE	NUE	NHE	NHI
**A. Dusty, 2004**					
1519-16 S	39.4A	0.49B	18.94BC	0.40B	0.82BC
1553-25 G	40.5A	0.48B	19.37BC	0.41B	0.84AB
1584-12 G	40.0A	0.47B	18.77C	0.39B	0.85A
Scarlet	40.9A	0.48B	19.92BC	0.40B	0.81C
3512-26 T	39.9A	0.51AB	20.26B	0.43AB	0.84AB
3586-6 G/T	39.4A	0.47B	18.75C	0.39B	0.82BC
3512-1 G	40.3A	0.50AB	20.32B	0.42B	0.84AB
Tara	40.0A	0.56A	21.96A	0.47A	0.84AB
**B. Endicott, 2005**					
1519-16 S	33.1C	0.49B	15.36C	0.34B	0.71B
1553-25 G	35.8AB	0.48B	16.09C	0.33B	0.73AB
1584-12 G	33.4BC	0.49B	16.08BC	0.36B	0.73AB
Scarlet	34.7ABC	0.52AB	17.56BC	0.37B	0.72AB
3512-26 T	36.1A	0.50B	17.85BC	0.37B	0.75A
3586-6 G/T	35.1ABC	0.60A	20.71A	0.44A	0.74AB
3512-1 G	35.4ABC	0.52AB	18.08AB	0.37B	0.72AB
Tara	36.0AB	0.50B	18.14AB	0.37B	0.74AB

Same letters following means represent non-significant differences within a site-year according to the Least Significant Difference test (alpha = 0.05).

**Table 4:** N rate means averaged over all genotypes for N utilization (NUTE), N uptake (NUPE), nitrogen use efficiency (NUE), grain N harvest efficiency (NHE), and N harvest index (NHI).

N Rate (kg/ha)	NUTE	NUPE	NUE	NHE	NHI
**A. Dusty, 2004**					
0	44.4A	0.76A	32.5A	0.66A	0.87A
45	45.5A	0.49B	22.0B	0.42B	0.87A
90	44.3A	0.41C	17.7C	0.35C	0.86A
135	37.0B	0.40C	14.6D	0.32C	0.81B
179	28.7C	0.42C	11.9E	0.32C	0.77C
**B. Endicott, 2005**					
0	43.3B	0.62A	26.3A	0.48A	0.78A
45	46.6A	0.43C	19.6B	0.33C	0.78A
90	36.9C	0.52B	18.2B	0.38B	0.74B
135	29.2D	0.48BC	13.2C	0.334C	0.71C
179	21.2E	0.49BC	9.9D	0.30C	0.64D

Same letters following means represent non-significant differences within a site-year according to the Least Significant Difference test (alpha = 0.05).

The NUE averaged over all genotypes decreased with increasing applied N for both site-years, attributable to decreases in both NUPE and NUTE (**Table 4**). Similarly, NHE also decreased with increasing N rate, most attributable to decreased NUPE, and to lesser extent, reduced NHI (**Table 4**).

### Tradeoffs Between Grain Protein Production and Unused Reactive Soil N

**Figure 3:**
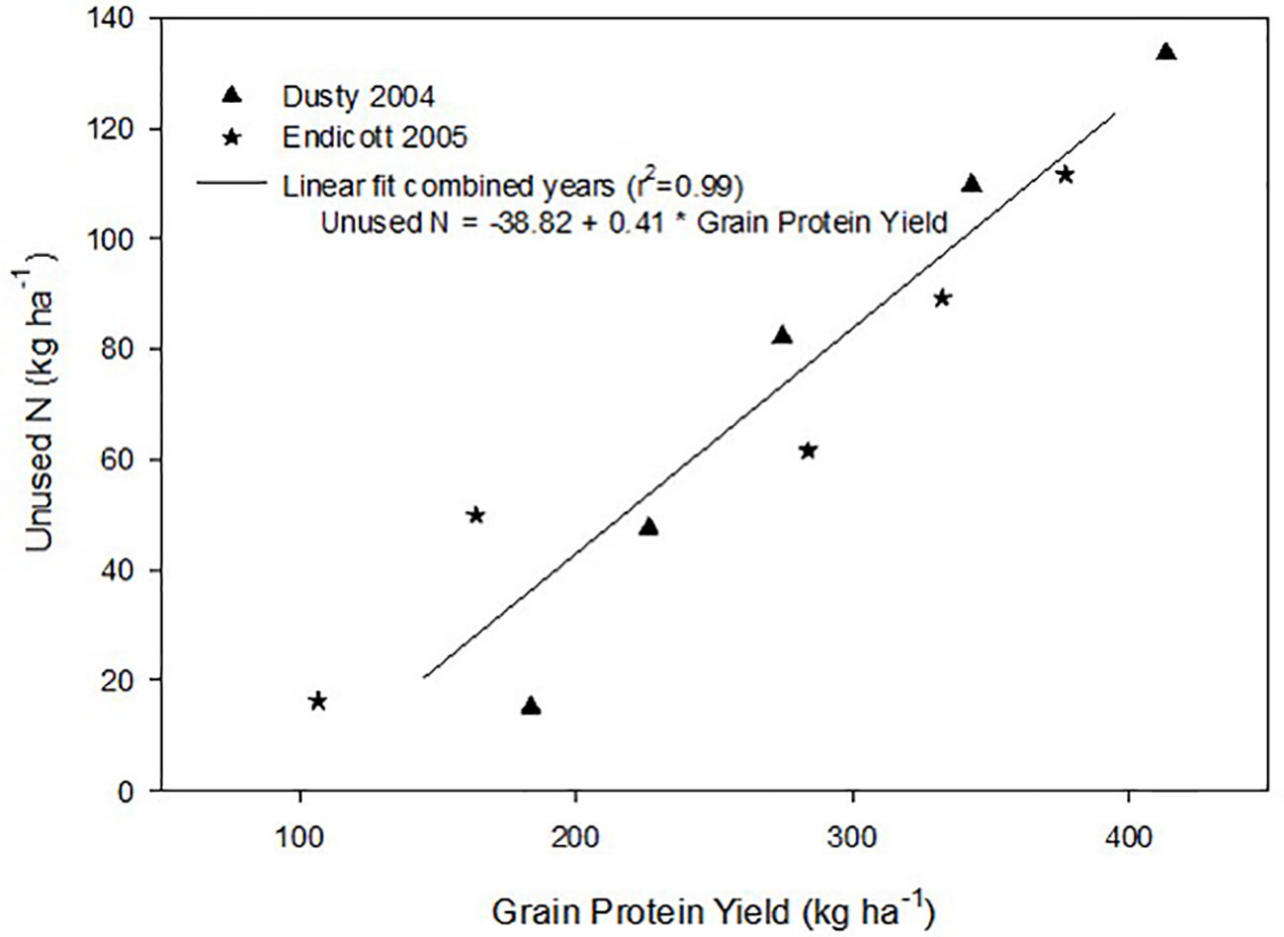
Linear relationship between the amount of unused soil N left behind after harvest and the grain protein yield produced at each N supply over two years. Mean data averaged over all genotypes and replicates in both site years are linearly regressed.

Unused reactive soil N left behind after harvest was calculated as the difference between N supply and crop N accumulation. A linear relationship between grain protein harvested and unused reactive N was observed in both site-years, without significant N supply x site-year interaction (**Figure 3**). Unused N increased by 0.41 kg N ha^-1^ per 1 kg protein ha^-1^ increase.

## Discussion

Re-cropping hard red spring wheat after winter wheat replacing fallow is a strategy for crop intensification for diversifying the system and markets (Pan et al., 2017). However, this shortens the time of soil N mineralization that would otherwise add greater available mineral N during fallow. For example, diminished fertilizer N responses of canola were earlier observed following fallow in this region (Pan et al., 2016). Steeper initial yield responses to N fertilizer inputs were observed herein with recropped HRS, with diminishing returns with higher N inputs represented by the Mitscherlich model (**Figures 1** and **2**). The modest maximum grain yields (A values) were due to the low soil water and in-season precipitation following winter wheat compared to fallow in this transitional agroecological zone.

Before a season, farmers can impact the protein level of their wheat by genotype selection and management of nitrogen. Yield and nitrogen both impact profit, so an economically motivated farmer will apply N at rates that optimizes both yield and protein. Baker et al. (2004) found that it is not always profitable to use 14 g (100g)^-1^ as the base protein goal for fertilization. Depending upon the wheat price premium/discount and the cost of N, in some scenarios profit was greater with higher yield and lower than base protein levels.

The price of N fertilizer and the protein premium/discount were held constant at current levels to assess the economic (**Table 1**) and ecosystem (**Figure 3**) impacts from varying the N supply. The GPC ranged from 10.3 to 13.4 g (100g)^-1^ at solely yield-based EOYs (**Table 2**). The economic analysis that accounted for GPC premiums and discounts revealed greater economic returns from elevating N supply above that required to achieve the yield-based optimum (**Table 2**). Only Scarlet in the first site-year showed lower economic returns (**Table 2**) from raising the N supply to >300 kg N ha^-1^ necessary to achieve GPC of 14 g (100 g)^-1^ (**Figure 1**).

The *Gpc-B1* introgression has been associated with earlier flag leaf senescence (Uauy et al., 2006) and greater N remobilization, along with higher N harvest index (Kade et al., 2005) that promotes higher GPC. However, as Carter et al. (2012) suggested, physiological benefit may have limited potential for improving GPC where spring wheat grain-filling periods are already shortened by environmental conditions in the inland Pacific Northwest.

Brevis and Dubcovsky (2010) demonstrated that *Gpc-B1* introgression increased protein yield in common and durum wheat. In the present study, the physiological benefit was not observed at any level of N supply, from deficient to excessive. Varying N supply with the addition of fertilizer N within the two site-years had greater impact on protein, yield, N use and its components, and economic returns than introgression of the *Gpc-B1* allele in these two hard red spring wheat cultivars. However, advanced Scarlet lines generally had higher GPC than advanced Tara lines at EONS determined on base yield price only (**Table 2**).

Maximizing protein-based economic returns with increased N supply can incur an environmental cost, demonstrated by decreased N use and its components with increased N supply (**Table 4**), as previously observed (Huggins and Pan, 1993). Application of fertilizer N required to produce >400 kg protein ha^-1^ also left >130 kg unused N ha^-1^ (**Figure 3**), representing increased reactive N remaining in the system that has potential for negatively impacting the environment. The presence of greater reactive N requires an N management accounting and reduction of fertilizer N inputs in the next crop cycle to avoid reactive N losses to the environment (Schlesinger, 2009; Snyder et al., 2014). Field-performance and grain-quality based selective breeding lead to the release of Tara (Kidwell et al., 2002) that improved the economic returns on N investments compared to the older Scarlet cultivar. These results stress the importance of further developing genotypes with increased yield and GPC potential. While the *Gpc-B1* introgression did not further improve economically optimal yield and GPC of these hard red spring cultivars grown under these conditions, future research should further investigate new genotype × environment × soil interactions for improving N use efficiency, grain quality, and economic returns, while reducing reactive soil N.

## Data Availability Statement

The datasets analyzed in this article are not publicly available. Requests to access the datasets should be directed to WP, wlpan@wsu.edu.

## Author Contributions

WP prepared the initial journal manuscript, supervised data collection, analysis, and interpretation. KK developed the wheat genotypes, supervised field experimental design and maintenance, and manuscript editing. VM conducted economic analysis and manuscript editing. RB conducted experimental layout, soil and plant sample collection, Mitscherlich modelling and analysis of variance statistical evaluation. MA, graduate research assistant, organized literature review, initial methods description and draft dataset.

## Funding

The authors thank the following sources of support: USDANIFA Award #2011-68002-30191 from the USDA National Institute of Food and Agriculture, USDA National Institute of Food and Agriculture, Hatch project 1014527.

## Conflict of Interest

The authors declare that the research was conducted in the absence of any commercial or financial relationships that could be construed as a potential conflict of interest.
