# Supplementary material for: Economically Optimal Wheat Yield, Protein and Nitrogen Use Component Responses to Varying N Supply and Genotype
Source: Front Plant Sci. 2020 Feb 25;10:1790. doi: 10.3389/fpls.2019.01790 (PMC7052120; doi:10.3389/fpls.2019.01790)
Supplement: Supplementary file 1 [file DataSheet_1.pdf]

Table S1. Quadratic model coefficient of determination (r2), correlation coefficients a, b, and c for crop N, grain N yield, grain protein concentration, and response to N supply of all genotypes in each site-year shown in Figures 1 and 2. Coefficient significance: ᵻP≤0.1, *P≤0.05, **P≤0.01 of committing type I error.

	Model fit	Quadratic Coefficients					
		a (y-intercept)		b (linear)		c (quadratic)	
2004 Crop N	r^2	Coefficient	P value	Coefficient	P value	Coefficient	P value
1519-16S	0.86	5.99	NS	0.35	*	0.0003	NS
1553-25 G	0.83	6.36	NS	0.38	**	0.0000	Ns
1584-12 G	0.82	3.01	NS	0.58	**	-0.0011	*
Scarlet	0.87	4.49	NS	0.47	**	-0.0004	NS
3512-1 G	0.72	3.14	NS	0.61	**	-0.0012	NS
3512-26 T	0.84	0.00	NS	0.47	**	-0.0004	ᵻ
3586-6 G	0.85	6.10	NS	0.34	**	0.0002	NS
Tara	0.76	9.41	NS	0.45	*	0.0003	**
2005 Crop N							
1519-16S	0.83	0.69	NS	0.39	*	0.0007	NS
1553-25 G	0.79	1.97	NS	0.30	NS	0.0011	NS
1584-12 G	0.93	-0.56	NS	0.58	**	-0.0007	NS
Scarlet	0.85	2.67	NS	0.47	**	0.0000	NS
3512-1 G	0.93	-1.45	NS	0.63	**	-0.0007	NS
3512-26 T	0.94	-0.06	NS	0.51	**	-0.0001	NS
3586-6 G	0.90	6.25	NS	0.49	**	-0.0001	NS
Tara	0.87	0.15	NS	0.56	**	-0.0005	NS
2004 Grain N Yield							
1519-16S	0.81	25.68	*	0.04	NS	0.0008	NS
1553-25 G	0.70	30.79	*	-0.02	NS	0.0009	NS
1584-12 G	0.63	17.14	NS	0.26	NS	-0.0002	NS
Scarlet	0.69	21.86	*	0.17	NS	0.0001	NS
3512-1 G	0.40	17.79	NS	0.31	NS	-0.0005	NS
3512-26 T	0.68	29.17	*	0.07	NS	0.0006	NS
3586-6 G	0.78	27.90	*	-0.02	NS	0.0010	NS
Tara	0.58	44.82	**	-0.13	NS	0.0012	NS

Table S1 continued

	Model fit r^2	Quadratic Coefficients					
		a (y-intercept)		b (linear)		c (quadratic)	
		Coefficient	P value	Coefficient	P value	Coefficient	P value
2005 Grain N Yield							
1519-16S	0.91	-3.49	NS	0.46	**	-0.0006	NS
1553-25 G	0.94	-2.16	NS	0.43	**	-0.0006	ƚ
1584-12 G	0.78	-3.19	NS	0.53	**	-0.0011	ƚ
Scarlet	0.77	9.92	NS	0.26	NS	-0.0001	NS
3512-1 G	0.96	1.34	NS	0.40	**	-0.0004	NS
3512-26 T	0.92	-3.50	NS	0.51	**	-0.0008	ƚ
3586-6 G	0.83	18.04	*	0.20	NS	0.0003	NS
Tara	0.91	-4.35	NS	0.57	**	-0.0012	**
2004 Protein Concentration							
1519-16S	0.71	13.84	**	-0.07	*	0.0004	**
1553-25 G	0.60	13.23	**	-0.05	NS	0.0003	**
1584-12 G	0.62	12.79	**	-0.04	NS	0.0002	*
Scarlet	0.50	11.68	**	-0.02	NS	0.0001	NS
3512-1 G	0.70	13.74	**	-0.06	*	0.0003	**
3512-26 T	0.64	13.18	**	-0.05	NS	0.0003	*
3586-6 G	0.74	13.14	**	-0.06	*	0.0003	**
Tara	0.54	13.99	**	-0.06	NS	0.0003	*
2005 Protein Concentration							
1519-16S	0.81	11.36	**	-0.03	NS	0.0003	*
1553-25 G	0.82	10.22	**	-0.03	NS	0.0003	*
1584-12 G	0.82	11.78	**	-0.04	NS	0.0003	**
Scarlet	0.75	11.49	NS	0.49	**	-0.0001	NS
3512-1 G	0.84	9.47	**	-0.01	NS	0.0002	**
3512-26 T	0.89	10.07	**	-0.03	NS	0.0003	**
3586-6 G	0.75	12.31	**	-0.05	NS	0.0004	**
Tara	0.83	9.10	**	0.00	NS	0.0002	ƚ

Table S2. Analysis of variance of dependent variables of year, N rate, genotype and N rate x genotype interaction for crop parameters, N use and components: [ƚ]P≤0.1, *P≤0.05, **P≤0.01 of committing type I error.

Variable	grain yield	GPC	crop N	grain N yield	NUE	NUPE	NUTE	NHI
year (Y)	**	**	**	**	**	**	**	**
N rate (N)	**	**	**	**	**	**	**	**
genotype (G)	**	*	ƚ	**	**	NS	NS	*
NxG	NS	NS	NS	NS	NS	NS	NS	*
